# Housing for Women in Midlife and Older Who Have Experienced Domestic Violence

**DOI:** 10.1177/10778012251351915

**Published:** 2025-06-19

**Authors:** Lori E. Weeks, Kathleen Allen, Zihui Wang, Christie Stilwell, Opeyemi Adeyi, Arezoo Mojbafan

**Affiliations:** 1 School of Health Administration, 3688 Dalhousie University, Halifax, NS, Canada; 2 Muriel McQueen Fergusson Centre for Family Violence Research, 3427 University of New Brunswick, Fredericton, NB, Canada; 3 Faculty of Science, Department of Psychology and Neuroscience, 3688 Dalhousie University, Halifax, NS, Canada; 4 Faculty of Health, 3688 Dalhousie University, Halifax, NS, Canada; 5 School of Nursing, 3688 Dalhousie University, Halifax, NS, Canada

**Keywords:** ageing, women, housing, intimate partner violence, IPV

## Abstract

Few services exist that are specifically targeted at older women who have experienced intimate partner violence (IPV). In this research, 23 women residing in permanent not-for-profit housing designed for women who have experienced IPV participated in an interview. The overarching themes identified were: (1) It's not perfect, but it is safe, affordable, and permanent; (2) Supporting empowerment and meaningful contributions; (3) Experiences living with other women who have experienced IPV; and (4) Support from housing staff. This research contributes to the paucity of evidence about permanent housing for this population and will inform the further housing development.

## Background

Violence against women (VAW) is a global public health concern, and intimate partner violence (IPV) is one of the most prevalent forms of VAW across the globe (WHO, 2014). Intimate partner violence (IPV) refers to any form of harm, abuse, or violence (e.g., physical, sexual, psychological, financial) or coercive or controlling behavior that is perpetrated by an intimate partner or an ex-partner (WHO, 2014). Long-term IPV has been associated with numerous long-term physical and psychological health conditions among women of all ages and backgrounds (Fedina, 2024). Commonly reported long-term health conditions resulting from lifelong IPV include chronic pain, cardiovascular problems, gastrointestinal problems, neurological problems, respiratory problems, and sexual and reproductive health problems (Fedina, 2024). IPV has also been associated with various psychological and mental health problems including anxiety, depression, suicidality, post-traumatic stress disorder, sleep disorders, and substance use disorders (Fedina, 2024). IPV can also negatively impact women's social lives and identities, family structures, and their socioeconomic status and living conditions (i.e., housing) (Daoud et al., 2016; Kaufman, 2024; McGarry & Bowden, 2017; Stockman et al., 2015).

### Intimate Partner Violence Against Women in Midlife and Older

Women of all ages are at risk of being exposed to, and affected by, IPV. For this study, we will focus on women in midlife and older (approximately age 50 and older) who have experienced IPV. We focus on midlife and older women because of the age and gender-related vulnerabilities of this age cohort and their unique IPV-related needs and preferences (Weeks, 2020; Weeks et al., 2018, 2021). Midlife is generally viewed as spanning approximately age 40–60 (Infurna et al., 2020). It is important to study IPV in midlife because this is a time of transition in women's lives during which they may be juggling multiple caregiving responsibilities (e.g., grandchildren, adult children, and aging parents), focusing on career development and retirement planning, and enduring changes to their family leadership and health status (e.g., developing chronic illnesses) (Infurna et al., 2020). It is also important to study the experiences of older women (i.e., approximately age 60 and older) because prolonged exposure to IPV and can lead to severe long-term health consequences that may be exacerbated by frailty and social isolation (Pathak et al., 2019).

Over the last decade, there has been an emergence of research evidence on the prevalence and incidence of IPV among midlife and older women. Lifetime prevalence rates of IPV among women aged 50 and older range from approximately 16% to over 60%, depending on the type of violence examined and the population under study (Andrade et al., 2023; Kavak et al., 2018; Metheny & Essack, 2020; Sasseville et al., 2022). Psychological violence is the most commonly reported form of IPV against older women, with rates ranging from 10% to over 50% (Andrade et al., 2023; Giraldo-Rodriguez & Agudelo-Botero, 2024; Guedes et al., 2016; Mellar et al., 2023). Physical violence is reported by 1%–20% of older women, and rates of sexual violence are typically below 5% in population-based studies, though higher in clinical and qualitative samples (An et al., 2019; Andrade et al., 2023; Metheny & Essack, 2020; Rodriguez Hernandez & Esquivel-Santovena, 2020). Additionally, studies reported rates of approximately 1%–18% of recent or past-year IPV among older women (Cations et al., 2022; Dichter et al., 2018; Miszkurka et al., 2016). Psychological abuse remains the most commonly reported form of IPV, while physical and sexual violence tends to decline with age (Barbier et al., 2022; Pathak et al., 2019). Women in their 50s and early 60s report higher IPV rates than women 70 and older, though this pattern is not universal across all forms of violence or cultural contexts (Barbier et al., 2022; Jud et al., 2023; Metheny & Essack, 2020). Additionally, it is generally recognized that, like other types or forms of abuse, IPV among older women is underestimated (Fileborn, 2017).

While it is clear from the above evidence that many older women experience IPV, research suggests that family violence services are not always able to accommodate older women's age-related needs, such as complex medical needs and physical limitations (Altman, 2017; Crockett et al., 2015; LeBlanc & Weeks, 2013; McGarry et al., 2014). Service providers supporting abused older women do not always understand the complexity of presentations of violence in older women or even recognize that older women are experiencing IPV (McGarry et al., 2017). Similarly, elder abuse service providers are often not equipped to support women from both a gendered and relational lens (Weeks et al., 2018) and lack to take into account power relations (Straka & Montminy, 2006). Older women may experience IPV differently than younger women and thus face unique barriers in accessing help due to physical or cognitive health challenges and financial dependence on their partners (Meyer et al., 2020; Pathak et al., 2019). Scholars argue that service providers need to make adaptations to reduce barriers that hinder older women from seeking refuge from IPV and to prevent older women from returning to their abusive relationships (Kilbane & Spira, 2010; Shiel, 2016; Straka & Montminy, 2006).

Compared to services for younger women, there are few interventions that exist to specifically support women in midlife and older who have experienced IPV (Crockett et al., 2015). Existing services can be grouped into four types: (1) individual in-person counseling and support; (2) individual help over the phone, crisis lines, and helplines; (3) educational and/or therapeutic support provided in a group setting; and (4) short- and longer-term shelters and housing (Weeks et al., 2021). The remainder of this literature review will focus on the short and longer-term shelters and housing options for midlife and older women who experience IPV.

### Housing Options for Midlife and Older Women Who Experience IPV

The availability of adequate housing is critical for women's ability to flee an abusive relationship (Clough et al., 2014) and for stabilizing their health (Daoud et al., 2016). The most common form of housing support that is available to women fleeing an abusive relationship are emergency shelters and/or transition houses, and second-stage houses (Rempel et al., 2024). Emergency shelters and transition houses offer temporary housing for survivors of IPV, typically lasting days, weeks, or a few months (Maki, 2018). Second-stage houses provide longer-term accommodation to women who have left an abusive relationship but require continued support and safety and typically house women for 1–2 years (Maki, 2018). Research demonstrates that both shorter-term and longer-tern housing programs offer lifesaving services (Maki, 2023), address homelessness of women and children (Baker et al., 2010), and have positive outcomes for women (Yakubovich et al., 2022). However, women face systemic barriers to obtaining and utilizing these forms of housing, such as limited shelter space and housing policies that are inequitable and re-victimize women who have experienced IPV (Clough et al., 2014; Gezinski & Gonzalez-Pons, 2021; Rakus & Singleton-Jackson, 2024; Wilson & Laughon, 2015).

There is evidence that older women utilize emergency shelters (LeBlanc & Weeks, 2013; Lundy & Grossman, 2009; Weeks et al., 2016). However, there is a need for more accessible housing options for midlife and older women who have experienced IPV (Altman, 2017; Hightower et al., 2006; Roberto et al., 2013) that accommodate their needs, such as more private spaces, assistance with medications, group support with women of similar ages and life circumstances, and an accessible environment (LeBlanc & Weeks, 2013). There are some emergency shelters and short-term supportive housing designed for women in midlife and older (James et al., 2015; Straka & Montminy, 2006). There are also innovations emerging to provide short-term shelter for older adults experiencing abuse in care facilities, such as the Weinberg Center Shelter in New York (Reingold, 2006; Solomon, 2020). All of these forms of support are important in meeting the individual needs and preferences of midlife and older women. While many older women wish to remain in their home, especially those with long-standing ties to their home and community (Weeks et al., 2016), others prefer to move to a different location. Although short-term and longer-term housing options are available throughout Canada for women who experience IPV (Maki, 2018, 2023), there is no published research about permanent housing options for women who have experienced IPV in Canada, and in particular permanent housing for women in midlife and older who have experienced IPV.

Atira Women's Resource Society (Atira) (https://atira.bc.ca/) is a Canadian non-for-profit organization that offers permanent housing options to women as part of their comprehensive service provision for women experiencing violence that operates in the greater Vancouver area in British Columbia, Canada, a province with high rental housing costs (Stewart & Cloutier, 2022). Atira is an intersectional feminist organization with a mission to support women and children affected by violence by offering safe and supportive housing and by delivering education and advocacy aimed at ending all forms of gendered violence. Atira's mission is guided by four values: (1) inclusive feminism—an understanding that women's experiences of oppressive institutions are interconnected (e.g., sexism, racism, ableism, classism); (2) women-centered—the programs invite and encourage women's collaboration and active participation; (3) harm reduction—an understanding that women's experiences of gender-based violence and systemic oppression is central to their use of substances; and (4) innovation—to encourage women's individuality, creativity, leadership, transparency and accountability. Atira currently offers many affordable housing programs designed for women, including shelters and transition houses, second-stage houses, supportive housing, and independent living housing. These housing facilities offer a range of services for women, and some facilities are designed for specific populations such as women who are First Nations, women struggling with substance use, and women who are in midlife and older. Two of Atira's transition houses and three of Atira's supportive housing buildings, many of which are single-room occupancy buildings, are designated for women in midlife and older (Whitzman & Hunt, 2021). The purpose of this research is to (a) learn about the experiences of women in midlife and older who have experienced IPV and who utilize permanent housing provided by Atira and (b) identify the strengths and challenges of their current housing.

In this qualitative study, we were guided by a critical gerontological perspective (Agger, 2013; Fay, 1987) that sees society as being composed of social structures that shape the lives of older adults, causing inequalities and stratification within the society. This perspective helps to examine how services are organized and provided for vulnerable people to inform ways that they can be improved. We were also guided by a strengths perspective that focuses on assets, competencies, resources, and capacity development in individuals, families, and communities (DeFrain & Asay, 2007; Saleebey, 2006). While a feminist lens is often used in research and interventions focused on the abuse of women and girls, this lens is often neglected by scholars and practitioners working with or for abused older adults. Thus, in this research, we use an intersectional lens through paying attention to multiple factors that affect the experiences of the study participants (e.g., age, gender, socioeconomic status, health status, ethnicity).

## Methods

We conducted this qualitative study in accordance with the consolidated criteria for reporting qualitative research (COREQ) checklist to ensure thorough reporting (Tong et al., 2007).

### Participant Recruitment

Criteria for inclusion in this study included women (i.e., any person who identifies as a woman, including transgender women) in midlife and older (approximately aged 50 and older) who currently live in permanent housing provided by Atira's Women Resources Society (Atira) in the greater Vancouver area in British Columbia, Canada. Participants lived at one of the following three Atira housing buildings: Margaret's Housing for Older Women (Maggie's), Sísele Housing for Women who are Older (Sísele), and Secord Housing for Women (Secord). Each of these programs provides safe and affordable housing for women who have experienced violence, but there are some differences between the staffing and structure of the homes. Maggie's is a 25-bed independent-living residential program for women aged 55 and older in Burnaby. Each suite has a private bathroom, and some meals are provided. There are a few full apartments with a kitchen, but most women use common cooking facilities. Maggie's does not have staff present overnight. Sísele is a 32-bed residential supportive housing program for women aged 45 and older in the Downtown Eastside area of Vancouver. Each suite has a private bathroom and kitchenette, and all meals are provided. This home has staff always present. Secord is a 28-bed residential supportive housing program for adult women. This home has shared bathroom facilities and common kitchens are available, but all meals are provided. This home has staff always present.

Information about the study was shared electronically by an Atira administrator to the manager of each of the homes. The administrator communicated with the first author about an appropriate time and date to arrive at each home. The manager arranged for a private place within each home to speak with potential participants individually about the study and to conduct the interviews. On the appointed days, staff at each home shared verbal and written information about the study to potential participants, and staff assisted in scheduling interviews for women who were interested. Prior to each interview beginning, the first author provided each potential participant with a hard copy of the informed consent form, gave a verbal summary of the contents of the consent form, gave each woman time to review the contents, and answered any questions the women had. The women were informed that the purpose of the study was to provide insights to Atira and other service providers about the future development of housing and services for women in midlife and older who have experienced violence. It was made clear to them that the interviewer and the other members of the research team were affiliated with a university and were not employed by Atira, but that anonymous information from the results would be shared with Atira management. Informed consent was obtained verbally and documented on the digital recording. All participants consented to being interviewed, and in each case chose to be interviewed immediately.

### Data Collection

Only the first author and the woman being interviewed were present during the interviews. Each woman was interviewed once, and no follow-up interviews were conducted. Questions asked included demographic characteristics of the participants and semi-structured open-ended questions about Atira services or programs utilized, other programs and services utilized, any issues they experienced in accessing and utilizing services or programs, and recommendations about how to better meet the needs of diverse women in midlife and older. The questions were not pilot-tested but were informed by prior published research along with the theoretical perspectives guiding this research to understand multiple factors influencing the women's experiences and their insights into how existing services can be improved. In addition, an Atira administrator provided input into the development of the interview guide (e.g., experiences utilizing other Atira services).

A total of 23 participants completed an interview from the three Atira buildings (Maggie's *n* = 9, Sísele *n* = 9, Secord *n* = 5) in November and December 2022. Interviewing continued until it was clear that no new key themes were emerging, and we were able to create rich, thick descriptions and meaningful data (Fusch & Ness, 2015; Guest et al., 2006). The interviews ranged from 10 to 51 min long (*M* = 27 min). The audio of each interview was digitally recorded, and a verbatim transcript was created. Transcription was facilitated by using Otter.ai to create a draft transcript which was then finalized by a team member who listened to each recording and edited the transcript. The participants were not provided with the opportunity to review their transcript. Field notes were not added to the transcripts or considered as part of the data analysis process.

### Data Analysis

We used an inductive reflexive thematic analysis approach for analyzing the interview data and paid attention to how the researchers influenced or shaped the resulting themes (Braun & Clarke, 2022). Thematic analysis is a form of pattern recognition for inductive coding (Fereday & Muir-Cochrane, 2006) useful for understanding influences and motivations related to how people respond to events (Luborsky, 1994). Data analysis involved data familiarization, generating initial codes, searching for themes, reviewing themes, and defining and naming themes that represent responses within the interviews (Braun & Clarke, 2022; Fereday & Muir-Cochrane, 2006).

Draft codes were identified by the first author and discussed with the research team. Further refinements were made until the codes were finalized. At least two team members independently coded nine (39%) transcripts and then came to consensus on the final coding through discussion. The remainder of the transcripts were coded by one team member. A total of five team members were involved in coding. There were 15 codes identified. Once coding was completed, the team identified overarching themes that emerged from the data. Data analysis was facilitated by using QSR International's NVivo12 Plus software which aids in organizing and analyzing qualitative data. If participants indicated during the informed consent process that they wished to receive a summary of the results either by e-mail or mail, this was sent to them.

### Ethics

This research received approval from the Health Sciences Research Ethics Board at Dalhousie University (approval # 2022-6291). All data collected was stored securely in password-protected systems. Team members shared research data through the secure OneDrive system to ensure the protection of the data. Participants were assured that they would remain anonymous in any reports of the results, but the name of the Atira services utilized would be identified. The women were interviewed in the building in which they lived (e.g., in a common room reserved for conducting private interviews), and they were aware that staff and other residents could potentially know who chose to participate in an interview. A participant number is used to refer to quotes along with the name of the building they lived in. Each person who participated in an interview received a $25 gift card to a national chain of coffee shops, including one woman who did not complete an interview due to health issues she was experiencing.

The first author facilitated the consent process with participants and conducted all the interviews. She is a full professor who identifies as a woman in midlife and is an experienced researcher on the topic of women in midlife and older who have experienced IPV. Her research has contributed to identifying the unique needs of diverse women in midlife and older and ensuring that appropriate services are developed to meet their needs (e.g., Weeks et al., 2016, 2021).

## Results

We first present the characteristics of the women interviewed followed by the four overarching themes that emerged that highlighted both the strengths and challenges of Atira's permanent housing from women's experiences. The four themes that emerged focused on the strengths and challenges of women's housing situation are as follows: (1) It's not perfect, but it is safe, affordable, and permanent; (2) Supporting empowerment and meaningful contributions; (3) Experiences living with other women who have experienced IPV; and (4) Support from housing staff. Quotes from women living across the three buildings are incorporated where appropriate to illustrate the findings. Quotes are attributed using the participant number and name of the building.

### Participant Characteristics

See Table 1 for a summary of the characteristics of the women interviewed. To assure anonymity of the participants, this information is not reported separately for the three homes, and only non-identifying information is included. The average length of time that the women lived in their current home was almost 5 years, and this ranged from 8 months to 11 years. Almost 40% of the women identified as white and were born in Canada. Four were born in another country and immigrated to Canada. Over a third identified as Indigenous or part-Indigenous. Most were either in the 55–64 (65%) or 65–74 (26%) age group. While almost half of the participants did not complete high school, almost 40% participated in higher education. Most participants identified as either divorced or separated (44%) or single (39%) and only one participant currently had a romantic partner. Nine identified having at least one child and four of them also mentioned being a grandparent.

**Table 1.** Participant Characteristics.

Characteristic	*n* = 23	%
Length of time in current home		
2 years or less	7	30.4
3–7 years	10	43.5
8–11 years	6	26.1
Ethnicity, place of birth		
White and born in Canada	9	39.1
Indigenous or part Indigenous	8	34.8
Immigrant to Canada	4	17.4
Other	2	8.7
Age (years)		
45–54	2	8.7
55–64	15	65.2
65–74	6	26.1
Education		
Less than high school	11	47.8
Finished high school	3	13.1
Training beyond high school	9	39.1
Marital status		
Divorced or separated	10	43.5
Single	9	39.1
Widowed	3	13.1
Currently has a partner	1	4.3

All but one participant identified having physical health problems and these included challenges related to the cardiovascular and circulatory system (e.g., heart problems, stroke, high blood pressure), digestive or excretory system and renal system, respiratory system, and musculoskeletal system (e.g., arthritis, osteoporosis, mobility challenges). Many participants also identified having issues related to their dental health, vision, and complications from prior injuries. Approximately 60% of the participants reported having current mental health problems including depression, bipolar disorder, anxiety, post-traumatic stress disorder, and challenges related to dealing with various forms of loss. While we did not specifically ask about addictions, about 20% of participants described themselves as a former or current drug user.

While we did not specifically ask about experience of IPV or other forms of violence experienced over their lives, many of the women shared these experiences during the interview process, as exemplified in the following two quotes: “There was always violence around me. So, I don't like violence” (21 Secord). “I came home to an empty house one day, from him getting out of jail and coming in and taking everything we owned” (3 Maggie's)

The women discussed various types of housing arrangements prior to moving to their current home including living with partners or other relatives, couch surfing, or being in hospital. Several mentioned living in subsidized housing in the past. Some of the women spent time in a correctional facility. About half of the women had lived in a short-term shelter, such as a women's shelter, and other forms of short-term housing. The women discussed various and often intersecting issues contributing to their prior precarious housing situation including issues related to employment, poverty, the lack of affordable and safe housing, physical and mental health challenges including addictions, and experiences in the criminal justice system. The prior housing situation for several women was disrupted due to interpersonal issues. This could include something that happened to a roommate or family members who were sharing the cost of a home, such as the breakdown of a relationship with a partner, illness, or death.

### It's Not Perfect, But It Is Safe, Affordable, and Permanent

This theme captured several key structural and intertwining elements that demonstrated the positive impact the Atira Housing program had on the participants. While the women each had a private room, most did not have their own full kitchen. Some of the buildings have a shared kitchen space and shared meals, others have individual meals provided, and some had a mix of these meal service approaches. While many women identified appreciating receiving any meals or food provided, others stated they would like access to their own kitchen, either because they did not like leaving their room if they were sick to use the shared kitchen, or they preferred to prepare their own food. One resident shared this was something they missed about previous housing, “I had a proper kitchen. You know, like a stove and an oven. Like I could bake and make my own meals. In here you've got this just little like a hot plate. You got two elements” (11 Sisele).

Even with the limitation of most of the women not having access to a full apartment with a kitchen, there were some key elements that were often lacking from prior housing but present in their current housing provided by Atira: safety, affordability, and permanence.

#### Safety

Many women identified that they felt safe living in their home, and this helped them feel in control of their lives. It was clear that many of the women did not always have this stability in their lives. There were many things that contributed to these feelings of safety including the intersections of age and gender in that only women were allowed to live in these buildings and the women were older (in Maggie's and Secord). While a few of the women preferred to live in a home with women of all ages, many provided reasons why it is a strength to include only women who are in midlife and older. They felt more compatible living with women of a similar age and felt that they had a greater understanding of each other and thus could be better companions for each other.

There should be more than one of these. This place is fantastic. I never knew that I was gonna come here and live happy. You know, when you are old, you are alone, you die alone … but here we companion each other. (2 Maggie's)

Additionally, many identified that women who are older prefer an environment that is quiet and calm. “It gives them time to heal. Time to get better. It's quiet. It's not confusing around here. We're all on. We all go to bed early” (3 Maggie's). “I think just a sense of respecting one another knowing that we’re all older, and it's just that I find that it's quiet” (11 Sisele).

Several women felt that the environment is safer with just older women due to fewer women using drugs, there is less stealing, and that there are fewer men visiting. Additionally, all the buildings had one secure entrance, and the women felt safe living in their building despite being located in Vancouver's Downtown Eastside. Safety was also enhanced through each woman having a private room, which was not always the case in their prior housing arrangements.

Yes, that is the main thing. I got my privacy, because in the former places, even though I got privacy, they'll just open the door, the landlord, just open the door. Yeah, you can lock my door and nobody's going to bother you. That is one thing that I like. (12 Sisele)

A participant living at The Secord indicated: “I never felt safer. It's a secure building. You can’t just walk in the door. So even though it's not in a great neighborhood, you feel comfortable here” (20 Secord).

#### Affordability

The women expressed a great deal of satisfaction with the amount that they paid for their housing. Many of the women reported paying $375 (CAD)/month, except for a few suites with full kitchens at Maggie's that were more expensive but were capped at a certain amount under market value. The women were quite aware of the high cost of housing in the Vancouver area, especially how the cost of renting in the open market has escalated in recent years. “Sure, I would like to have one bedroom (apartment), you know, apart from that I can’t afford it now … its so expensive everywhere” (18 Maggie's). A woman living at Sisele indicated: “I couldn’t do anything else. Because when you’ve got $375 for rent, where can you rent … I don’t think I’d find accommodation for what I have as nice as this” (11 Sisele).

In addition to having highly subsidized housing, the women also appreciated having few additional costs associated with housing. Some of the women identified expenses included in their rent, such as cable TV and internet. While not all the women had a computer in their suites, this was available in the building, such as in a common room. At Sisele and Secord, all the costs of food were included in the cost of rent. At Maggie's, the women were expected to purchase groceries and do their own cooking, but some meals were provided (e.g., breakfast). There were several strategies utilized to reduce the costs of food for the women living at Maggie's including making use of a food bank and working with local organizations who donated food.

#### Permanence

In contrast to various other forms of housing the women experienced, they could live in their current home permanently if they wished to do so and their needs could continue to be met. While some of the women indicated wanting to move to a different type of housing (e.g., a full apartment with a kitchen), most of the women interviewed expressed gratefulness to Atira for their current home where they can live permanently.

I’m very thankful for Atira and all they’ve done for me. They’ve been very good. Like they’ve never closed the door on me … It gives women the time to just slow down, stop, and then get their self worth back because they have stability. In their life, it's really important to be stable. If you're not in a stable place, you're not going to get better. (3 Maggie's)

A women living at Sisele added: “Yeah. I wasn't left homeless, no, thank goodness … and I'm grateful to have a place. I guess I should say I'm grateful to Atira” (13 Sisele).

### Supporting Empowerment and Meaningful Contributions

Several women felt that living in an Atira building contributed positively to their well-being in several ways. A key strength identified from the interviews was that the women felt that several aspects of Atira programs encouraged empowerment and social connection. Atira supports empowerment of women through encouraging the women to support each other, contribute in an unpaid capacity to the operation of the home, or receive training or paid positions within Atira. Being part of a network of women who work for Atira and/or live in Atira buildings helped to provide positive examples of how women can support each other. About a third of the women spoke about various paid and unpaid contributions they made to support the other women living with them or women using other Atira services. “It's just been a network of women, that positive network that you could feel you feel good being around, you feel empowered. You know, you feel like you're not alone. It's really nice” (3 Maggie's).

Many of the women spoke about paid positions that they currently or formerly held with Atira. Some worked as a peer support worker, and this included providing education about the COVID-19 vaccine and encouraging the women to get vaccinated. Peer support also included a variety of other tasks such as helping other women gain access to services. Some of the women also spoke about being paid to do extra cleaning in the home related to preventing the spread of the COVID-19 virus.

I got a job in the building. I don't go to it right now, but I had one and then they asked for it back. You know, because of COVID. I liked that because it keeps me busy … I was just going around, like cleaning and everything … just like doing doorknobs, railings. I like it, you get little extra money plus it kept me busy for two hours. And it made me feel better about myself. (8 Sisele)

The women who lived at The Secord could make dinners for the other women, and they were compensated for this contribution.

And then we have a cooking program in here … We started it ourselves, basically where we cook dinners. And we get paid for that. So that's a bonus … I cook once a week … So there's like five or six of us right now and then we cook just one day a week. (22 Secord)

A few of the women mentioned currently being in an Atira training program, such as training to become a peer support worker, or previously completing training provided by Atira. They looked forward to obtaining a paid position with Atira in the future. “And so right now with this employment program and training, I'm trying to pull myself out” (5 Maggie's).

Some of the women also spoke about informal work or things they did to support the other women living in the home or looking after the building and property. Several women mentioned that they help cook for the other women or help them access food and other items they needed. Some women contributed in other ways such as volunteering to help with gardening and yard maintenance. “You know, if I see some clothes, and somebody needs clothes, and that, I grab them for them. If they are not eating properly, I try to get them something healthy to eat” (21 Secord). A woman living at Sisele indicated: “I help a lot of people. And I used to help people go to the store. If they don't want to go, I will go for them. If they need help doing the cleaning up, I do that help” (10 Sisele). Finally, a woman living at Maggie's contributed:

So I take care of the leaves, and do the weeding. And I did all the rocks around all the trees and stuff out there. So I've done that. Me and my girlfriend. Yes, she lives here too. She's done that with me. (3 Maggie's)

### Experiences Living With Other Women Who Have Experienced IPV

Almost all the women talked about how they had positive relationships with at least some of the women who lived in their building. Some felt that there were more opportunities to get to know the other women in Atira housing compared to living in a traditional apartment building. The relatively small number of units and the design of common spaces helped to foster relationships between the women and some women reported feeling like the other women were family, “It sounds really corny, but we’re almost like family” (13 Sisele). Shared kitchens, dining rooms, and lounges provide opportunities for the women to meet each other. Not feeling alone was very important to several of the women. Many of the women shared similar experiences of violence throughout their lives and they were similar ages, these factors appeared to help support positive relationships. One woman living at Secord said that the older women took the younger women under their wing and that was positive for both age groups.

It really made a bond for me and that woman to know what she had been through. Not that any one is worse than the other. You know, but, but and the whole cause of, for women to support one another. Yeah. Where I probably wouldn't have had that if I was on my own. (1 Maggie's)

Although many women reported positive relationships with other women, some of the women identified that interpersonal conflict and abuse between the women existed, and several women recognized that mental health issues contributed to this negative behavior. The women adapted by trying to be supportive of other women who displayed abusive behavior, disengaging from certain people, using common spaces less or not at all, and spending a lot of time in their own suite or outside the building.

Yeah. I do not judge because some women talk to themselves here. You know, and they'll yell and stuff and they think stuff's going on. And it's not, but you just be supportive of them. And if you're not, you learn how to be because you can't stick out. You can't be a person that's, you know, causing problems, or you won't be in here. So if you don't have an understanding or acceptance, it'll be known right away. (3 Maggie's)

It was clear from the women's stories that many of the women living in Atira housing were current or former drug users. Several of the women did not want to be around people who were drunk or high. The negative impacts included being bothered by this behavior, noise, not feeling physically safe, and being reminded about situations they had to deal with in the past (e.g., relatives who abused drugs).

Some of them are still drug users and dealers. And some of us say no, and when we say no, we get attacked. You know, we get verbally, verbally attacked or verbally threatened physical, physical violence and they get I just think it would be best if we were all separated. I mean, like, I'm in my 60s now. (5 Maggie's)

Several participants brought up the recent death of a resident due to addictions, and this was difficult for the women to deal with. “She passed away, a week ago in the hospital, so we are missing her. And we are kind of quiet, a little bit changed” (4 Maggie's). Several women indicated that they agreed with the harm reduction philosophy in place including being provided with safe supplies and access to the services of a pharmacist. “They supply needles and pipes and things like that. Yeah, harm reduction … which is great, it's much needed” (15 Sisele).

The women either directly or indirectly identified practical suggestions to help with interpersonal conflict and abuse between residents. Several women identified that they did not have the skills to interact with people who are abusive, and training could be useful, such as how to interact with people having mental health issues (e.g., de-escalation techniques). House meetings could be useful to exchange information. Interventions with those exhibiting abusive behavior were identified as a solution. Some women expressed the sentiment that if a resident is being abusive toward other residents, that resident should have to leave the home, as shown in the quote below:

No one quite knows how to handle people with mental health issues … the changes being suggested are for us to change our behaviour, not for the bully or perpetrator. I don’t agree with that. We did enough adjusting, you know, we stayed in abusive relationships for years, because we didn’t know how to get out and there wasn’t people to help us. But we’re old now. We here to live a peaceful life in tranquility … I don’t think they should be allowed to live here, because it's not just bullying and racism, there's also elder abuse. (17 Maggie's)

### Support From Housing Staff

The women discussed relationships and support from Atira staff and administrators extensively, including both strengths and challenges. Many of the women noted that they have positive relationships and support from staff and administration in general, and this contributed to the benefits derived from living in Atira housing. Women's narratives revealed how the Atira staff supported the women in a variety of ways including: fostering safety and security, supporting women's physical health and medical needs, providing social support and mental health support, and helping women access information and services.

#### Fostering Safety and Security

Having staff present in the homes and available to help if an incident happens contributed to the women feeling safe and in control of what happens to them. This can include feeling safe from situations outside their home and helping with any issues that occur within the home, such as interpersonal issues. There were also mechanisms in place for the women to report incidents to the manager of the home or to the Atira administration, and this was appreciated by the women living in all three building. “They are there if you need them, you know, I like that” (11 Sisele) “You can live your life any way you want within reason, and you have that feeling of the staff helping” (21 Secord). “I’ve made sure that if there's anything wrong, I will right away send an e-mail to the program manager or something” (3 Maggie's).

#### Supporting Women's Physical Health and Medical Needs

Many of the women identified ways in which the staff and administration supported their health and medical needs. This included staff helping with scheduling medical appointments and providing reminders about upcoming appointments to women. Staff also helped to get women safely to an appointment, personally accompanying women to medical appointments ensured that any health information and instructions were recorded and monitored. “They walk us to the doctor's that is within walking distance. They walk us to appointments … or if you need a drive, or go with them on the bus” (14 Sisele).

I am really bad for going for appointments and stuff like that. So the staff take me to doctor's appointments, and they pay attention, cause I have seizures and memory loss. I might get too excited or too emotional, my body seems to shut down. Just like fall asleep or pass out from pain or whatever it is. And so they keep notes and stuff like that. (20 Secord)

#### Helping Women Access Services and Information

Women were very appreciative of the support that staff provided to them with helping them access information and other services. This included support with finding information online and helping to identify what services are available.

They have said if anyone is grieving (staff member) did say, to come to her, and she can help to get some counseling, and stuff like that, which is good. That's really good. Because people need that to be able to talk. (1 Maggie's)

And they will help you with Old Age pension, Canada pensions, or your medical service plan if you need anything, and they can find a way to help you. And if I was on my own, I believe me, I wouldn't even have a clue where to begin with most of that … Like if you wanted to speak to counselors, or psychologists or something like that. They would, they would suss out on how to get you there. (4 Maggie's)

#### Providing Social Support and Mental Health Support

Several women provided examples of how the staff provided them with guidance and social support. “They are very good guidance … and they are great just to talk to” (9 Sisele). Additionally, women living in all of the buildings discussed ways that staff supported their mental health struggles and provided social support.

Something that I have is depression. And when I'm not taking my meds, I'm really not very, in a very good way. So I have to take my medicine all the time… They see me in a bad mood. They will say did you take medicine. (3 Maggie's)

“There was also a staff person who was very good in, in kind of, like, helping me to calm down my mind, and focus” (1 Maggie's). “When I first came here, I was in really bad shape, you know, and it was the people here that helped keep me together” (11 Sisele).

#### Challenges With Staff

While there was more overall discussion about positive experiences with staff, some concerns identified by the women related to relationships with staff. It was not uncommon for the women to identify that they got along well with certain staff members more than others. “They’re actually really good staff that's here working now. We had a couple of bad eggs, but they’re gone” (23 Secord).

Some of the staff are unbelievably great. Super helpful, unbelievable great personalities and workers. Other staff are dead inside. That's how I'd have to call that … Well, the good staff, the ones that have great personalities. They do your laundry or clean your room or stuff like that, will help you in your room. But the dead staff of course, it's not in their job description … Other staff members are just fantastic. I feel well blessed. (15 Sisele)

Some women raised concerns about how some staff were not caring and were limited in the support they would provide, and staff who lack specific skills, such as cooking skills. Although the women realized that staff can not share confidential information, they felt staff should be able to let them know if any of the other women were ill and seeking medical treatment. A particular challenge at Maggie's was that there is no staff present overnight, and the hours of staff coverage during the daytime seems to have been reduced over time. The reduction in staff hours has had an impact on the ability of staff to prepare meals, especially dinner, and to support other activities. A few women discussed concerns related to staff turnover as this affected the relationships they had with staff. “Well, I think, you know, I think they are short staffed to be honest with you. So, you know, personally, I would be nice that if they can do it would be nice to have consistency” (16, Maggie's). “Like I was saying, with our staff, you start getting to know them? And then they ended up getting transferred somewhere else, and so like, now us ladies we're really picky on who we talk to now” (7 Sisele).

## Discussion

This study was guided by a critical gerontological perspective (Agger, 2013; Fay, 1987) and a strengths-based perspective (DeFrain & Asay, 2007; Saleebey, 2006) using an intersectional lens. Combining these perspectives allowed us to identify how Atira's services are positively impacting the participants’ lives and what aspects of the services can be improved to meet the needs of women in midlife and older who have experienced IPV. This study contributes importantly to our knowledge about the provision of affordable, safe, and permanent housing to women in midlife and older who have experienced IPV. Many older women who are low-income face housing affordability challenges, especially those who are renting in a large city (Stewart & Cloutier, 2022). Several elements in the housing the women in this study utilized are congruous with Canada's National Housing Strategy including homes that are safe and affordable and that housing investments must prioritize those in most need, including women who are fleeing IPV (Canadian Mortgage and Housing Corporation, 2018). Together, our findings highlight the importance of providing safe and stable (i.e., permanent) housing options with supportive staff and offering opportunities for empowerment.

While the private units were small and lacking some features, the women appreciated having a private space, and the services provided by staff were a key strength. There were concerns identified with the design of the homes, and especially that most did not have access to an apartment with a full kitchen. It is clearly a challenge for non-profit organizations to be able to provide highly subsidized housing that also include the amenities of a full apartment. The lack of a full apartment was balanced by the amenities and services offered in these homes. The combination of housing with supports appeared to be crucial for the women in this study. The integration of housing and services is integral in supporting ageing-in-place, which refers to an approach in which older adults are supported to live in their home and/or community with independence for as long as possible (Chum et al., 2022). Atira supported women to age-in-place in several ways including providing opportunities for women to build social relations, offering support and services that help women to prioritize their health and well-being, and fostering a sense of autonomy and empowerment by offering women paid and unpaid positions in their facility to give back to others in their residential community. The women appreciated any compensation they received for this work and also the appreciation received for unpaid contributions. It was evident that the women received personal rewards for making meaningful contribution to others. Assisting in the process of empowerment is an important part of effective interventions for older women who experience IPV (Tetterton & Farnsworth, 2011) and this aspect should be emphasized in the further development of future housing program for women in midlife and older who have experienced IPV.

The participants spoke about the positive experience of living with other women who are at a similar life stage and with similar experiences. Participants also highlighted various forms of support that they received Atira staff including fostering safety and security, supporting women's physical health and medical needs, providing social support and mental health support, and helping women access information and services. Recent research has revealed the importance of integrating social support into IPV interventions for improving mental health outcomes and for meeting women's age-related needs (Ogbe et al., 2020; Pathak et al., 2019; Safar et al., 2023). By offering both formal and informal support to older women Atira is promoting resiliency by offering opportunities for women to build social connections among women who are at a similar life stage and who have also experienced IPV (Howell et al., 2018). Fostering these connections helped reduce loneliness and social isolation among the participants, which is commonly experienced by older women experiencing IPV (Band-Winterstein & Eisikovits, 2005; Hing et al., 2021; Meyer et al., 2020).

Although Atira promoted ageing-in-place and resiliency in the women, some challenges were identified by participants. Some participants experienced difficult interpersonal relations with other women, such as those with mental health issues and/or addiction issues. This appeared to be particularly challenging for the women who wanted to distance themselves from others who were using abusing drugs. Although women believed that harm-reduction practices were important for women who were using drugs, it would be beneficial for Atira to explore ways to limit contact between those abusing drugs and those not abusing drugs, to ensure that women who are not using drugs feel safe and secure in their home. Forming partnerships between health care providers and organizations providing affordable housing can be advantageous in supporting residents who abuse substances, and especially opioids (Pollack et al., 2022).

Another challenge that participants grappled with was turnover among Atira staff members. Atira is not unique in their struggle to provide appropriate staffing in their housing and programming. Research has demonstrated that other family violence services (e.g., emergency shelters, second-stage housing programs) in Canada are also dealing with a high rate of staff burnout and staff turnover (Maki, 2019a, 2019b). Chronic underfunding has been identified as a barrier to providing adequate training, staff, and programming among family violence services in Canada (Maki, 2023). Working with survivors of violence also comes with the risk of staff experiencing vicarious trauma, compassion fatigue, and burnout (Baird & Jenkins, 2003). Given that some women described some staff and uncaring, it may be that staff are struggling with these issues (e.g., compassion fatigue). Offering education on self-care for staff is warranted.

We identified several limitations in this research. We interviewed women utilizing housing at three different sites provided by one Canadian non-profit organization operating in the greater Vancouver area, in British Columbia, Canada. Two of the homes were located in a neighborhood in the urban core of the city facing complex social issues (e.g., homelessness, poverty, drug use) and the other was in a suburban environment. In addition, the physical layout and services provided at each location varied slightly. This created some variations in the findings across the homes, but we included illustrations from women across the three homes included to provide evidence supporting each of our themes identified. In addition to funds received through user fees and fundraising, Atira receives funding from the provincial housing authority, British Columbia Housing (bchousing.org) in order to provide highly subsidized housing. Atira is also a social enterprise in which income from for-profit activities help to subsidize housing. Our results may not be transferrable to other cities in other locations that have different mechanisms for providing affordable housing.

Older women who leave an abusive partner face many barriers in the process of leaving, only one of which is accessing safe housing (Hightower et al., 2006; Roberto et al., 2013), and services need to be tailored to older women's unique needs (Pathak et al., 2019). Our findings reveal that Atira housing for older women is meeting the unique housing needs for many older survivors of IPV in the Vancouver region, in British Columbia, Canada. Given that this study was conducted in a large city we did not provide insights into the provision of housing for women in midlife and older living outside of urban areas, such as older women living in rural locations may have unique needs when seeking help (Weeks et al., 2016).

## Conclusions

Through our focus on the intersections of an age and gender-based feminist lens from a critical gerontological perspective (Agger, 2013; Fay, 1987), this research contributes to the paucity of evidence about permanent housing initiatives to support women in midlife and older who have experienced IPV (Weeks et al., 2021). This research provides important insights about the specific housing and support needs of women in midlife and older with a history of IPV. The common challenges experienced included living with other women who have mental health and/or addiction issues, interactions with unsupportive staff, and staff turnover. Although the women in this study identified ways to improve this form of housing, through a strengths-based perspective utilized in this research (DeFrain & Asay, 2007; Saleebey, 2006), we identified several strengths across the three homes including offering affordable, safe, and permanent housing, supporting ageing-in-place, promoting empowerment and resiliency, and reducing social isolation. This study also highlighted the importance of the bilateral relationship of both receiving social support and providing social support. Longitudinal research on the long-term outcomes (e.g., mental health, physical health, perceived social support) for women utilizing permanent supportive housing is warranted.

Given the little research evidence available about this form of housing for women in midlife and older who have experienced IPV, researchers should partner with additional organizations to share knowledge about the characteristics of innovative forms of housing and provide insights about the experiences of the older women using these services. Further research will be instrumental in informing the development of permanent supportive housing for women in midlife and older who have experienced IPV in various jurisdictions.

To conclude, we argue that Atira's not-for-profit organization offers a novel permanent supportive housing program to women in midlife and older who have a history of IPV in British Columbia, Canada. Atira provides valuable supportive housing and services to aging women that extend beyond the traditional housing programs provided in the family violence sector (e.g., emergency shelters, second-stage housing), that can serve as a model for other organizations.
